# Relationship Between Educational Level and Attitudes Towards Alcohol Conversations in Healthcare: A Cross-Sectional Survey Conducted in Four European Countries

**DOI:** 10.3389/ijph.2023.1605634

**Published:** 2023-03-24

**Authors:** Nadine Karlsson, Janna Skagerström, Amy O'Donnell, Latifa Abidi, Kristin Thomas, Per Nilsen, Torgeir Gilje Lid

**Affiliations:** ^1^ Department of Health, Medicine and Caring Sciences, Linköping University, Linköping, Sweden; ^2^ Research and Development Unit in Region Östergötland, Linköping, Sweden; ^3^ Population Health Sciences Institute, Newcastle University, Newcastle upon Tyne, United Kingdom; ^4^ Department of Health Promotion, Maastricht University, Maastricht, Netherlands; ^5^ Centre for Alcohol and Drug Research, Stavanger University Hospital, Stavanger, Norway; ^6^ Faculty of Health Sciences, University of Stavanger, Stavanger, Norway

**Keywords:** prevention, healthcare, alcohol, socioeconomic factors, population survey, attitude, perceived honesty, trust

## Abstract

**Objectives:** To examine the association between educational level and attitudes towards alcohol conversations in healthcare using population-based surveys of adults in England, the Netherlands, Norway, and Sweden; and to compare attitudes towards alcohol conversations in healthcare between these four countries.

**Methods:** Cross-sectional surveys were conducted amongst adults in the general population in England (*n* = 3,499), the Netherlands (*n* = 2,173), Norway (*n* = 1,208), and Sweden (*n* = 3,000). Logistic regression analysis was used to examine associations between attitudes towards alcohol conversations in healthcare and educational level, key demographic variables, alcohol consumption, and country of residence.

**Results:** In all four countries, low educational level (*p* < 0.001) and male gender (*p* < 0.001) were associated with holding negative attitudes towards discussing alcohol in healthcare. Risky drinkers had more negative attitudes than low risky drinkers towards discussing alcohol in healthcare (*p* < 0.001) in all countries except England (*p* = 0.48), and also reported low levels of perceived honesty and confidence in healthcare (*p* < 0.001).

**Conclusion:** These findings highlight the importance of considering patients’ socio-economic status when developing and implementing alcohol prevention interventions in healthcare.

## Introduction

Alcohol is a key global risk factor for morbidity and premature mortality, and an important cause of both intentional and unintentional injuries (1), making reduction of heavy drinking a critical public health goal (2). Harmful alcohol use is also associated with significant adverse social and economic outcomes that extend beyond individuals to society as a whole (3). However, research suggests that these harms are not evenly distributed across populations. Specifically, there is substantial evidence that alcohol-related morbidity and mortality are more commonly experienced by those in lower socio-economic groups (4), despite the fact that overall they report drinking the same or less than those from higher socio-economic groups: a phenomenon known as the “alcohol harm paradox” (5).

Brief alcohol interventions (BI) delivered in primary care have proven to be effective for the prevention of hazardous and harmful drinking (6). There has been limited exploration to date of their potential contribution to addressing alcohol-related health inequalities, although findings from one recent English study suggest that disproportionately high rates of BI delivery in lower socio-economic groups may be having a positive impact in terms of reducing harmful drinking (6, 7). Socioeconomic status (SES) is commonly conceptualised as a combination of economic, social, and work status (8), and measured by education, income/wealth, and occupation respectively. Previous research has shown that there is an association between SES and health outcomes (9). There is also evidence that SES and certain demographic characteristics can shape patient participation in and beliefs about healthcare (10), with lower health literacy, differences in role expectations, and previous experience, cited as contributing factors. However, we have little understanding of how socioeconomic position might influence attitudes towards alcohol conversations in primary healthcare.

Findings from one systematic literature review showed that socioeconomic position influences the acceptability, attendance and outcome of alcohol BI in primary healthcare (11). A population-based survey conducted in Sweden in 2011 found low levels of support for routinely asking patients about their alcohol consumption in healthcare amongst younger, less educated drinkers (12). Previously published studies from this research group have studied the association between patient characteristics and attitudes towards and experience of alcohol conversations in healthcare in England (13), the Netherlands (14), Norway (15, 16) and Sweden (16, 17), and showed an association in England between low SES and negative attitudes towards alcohol conversations in healthcare (13). All four countries are relatively high income countries located in Western Europe, and share many similarities in alcohol consumption patterns (18). However, there are country-level factors that might influence attitudes towards alcohol conversation in healthcare, for example, government alcohol policy, the role of alcohol industry, and the socio-economic distribution of alcohol-related harm (19)*.*

To date, however, no study has compared and investigated the association between SES and attitudes towards alcohol conversations in all four countries.

With evidence of increasing health inequalities throughout Europe (20), cross-national analysis is needed to better understand whether and how SES is related to attitudes towards alcohol prevention efforts in different national contexts. This study aimed to examine the association among adults between educational levels and attitudes towards alcohol conversations in healthcare using population-based surveys in England, the Netherlands, Norway, and Sweden, and to compare patterns of attitudes towards alcohol discussion in healthcare between these countries. We used education as a proxy measure of socioeconomic status. Education is a measure of cognitive skills, such as information-gathering, that are necessary to make informed decisions that promote health (21). Education is also a result of other individual and contextual resources. A range of data confirm that people with a higher level of educational attainment tend to have better health and educational differences in health behaviours (physical activity, smoking, risky drinking) are major drivers of health disparities (22). By comparing these four countries, we sought to gain a better understanding of how context may influence attitudes amongst different socio-economic groups. In turn, this will enable the development of improved strategies to tackle the higher alcohol-related morbidity and mortality in lower socio-economic groups in future.

## Methods

### Study Population and Design

Population-based cross-sectional online surveys of adults were conducted in 2017 in England (*n* = 3,499) (13), the Netherlands (*n* = 2,173) (14), Sweden (*n* = 3,000) (17) and in 2018 in Norway (*n* = 1,208) (15, 16) (Table 1). Data on socio-demographic characteristics, educational level, alcohol consumption, and attitudes towards and experiences with strategies for addressing alcohol in routine healthcare were collected. Individuals aged 18 years or older were eligible for inclusion in all countries other than England where the lower age limit was 16 years (13). Data collection methods specifying details for each country are described in previously published articles (13–17).

**TABLE 1:** Sample characteristics by country. England, Netherlands, Norway, and Sweden, 2017–2018.

Variables	Country			
	England	Netherlands	Norway	Sweden
Gender	3,499	2,173	1,208	2,996
Man	1,736 (49.6%)	1,015 (46.7%)	551 (45.6%)	1,501 (50.1%)
Women	1,763 (50.4%)	1,158 (53.3%)	657 (54.4%)	1,495 (49.9%)
Age (in 5 categories)	3,471	2,173	1,208	3,000
≤29 years	720 (20.7%)	316 (14.5%)	165 (13.7%)	851 (28.4%)
30–39 years	486 (14.0%)	259 (11.9%)	208 (17.2%)	604 (20.1%)
40–49 years	506 (14.6%)	296 (13.6%)	240 (19.9%)	630 (21.0%)
50–59 years	554 (16.0%)	390 (17.9%)	237 (19.6%)	591 (19.7%)
≥60 years	1,205 (34.7%)	912 (42.0%)	358 (29.6%)	324 (10.8%)
Education	3,196	2094	1,191	3,000
Basic or secondary school	2,153 (67.4%)	783 (37.4%)	356 (29.9%)	1,533 (51.1%)
University	1,043 (32.6%)	1,311 (62.6%)	835 (70.1%)	1,467 (48.9%)
Drinking categories^a^	3,484	2,172	1,208	2,996
Low risky drinking	3,013 (86.5%)	1,526 (70.3%)	839 (69.5%)	2,149 (71.7%)
Risky drinking	471 (13.5%)	646 (29.7%)	369 (30.5%)	847 (28.3%)
Weekly alcohol consumption (in g)				
Mean (SD)	55.6 (96.3)	56.6 (76.6)	42.2 (61.9)	42.1 (66.3)
Median (IQR)	16 (80)	22.5 (78)	18 (43)	18 (43)

^a^ Different definitions used in England, Netherlands, and Norway/Sweden—see methods.

### Measures

#### Attitudes Towards Alcohol Conversations in Healthcare

Attitudes to being asked about alcohol in routine healthcare were investigated using five questions (see Table 2), with possible response options of “agree completely,” “agree to a large degree,” “agree to a small degree,” and “do not agree” to each question (12).

1. Healthcare providers (physicians, nurses, etc.) should routinely ask about patients’ alcohol consumption (“routine”).
2. Alcohol consumption is a personal matter and not something healthcare providers should ask about (“private”).
3. Healthcare providers should ask about patients’ alcohol consumption, but only if patients seek healthcare to discuss symptoms that could be related to high alcohol consumption (“symptoms”).
4. Healthcare providers should ask about patients’ alcohol consumption, but only if the issue is brought up by the patient (“patient”).
5. I believe people answer honestly when they are asked about their alcohol consumption at healthcare visits (“honesty”).

**TABLE 2:** Attitudes about alcohol prevention by country. England, Netherlands, Norway, and Sweden, 2017–2018.

	Total, *n* (%)	Country				*p*-value
		England	Netherlands	Norway	Sweden	
(1) Healthcare providers should routinely ask about patients’ alcohol consumption (“routine”)	9,834					
Do not agree	2,300 (23.5%)	421 (12.2%)	323 (14.9%)	106 (8.8%)	263 (8.8%)	<0.001
Agree to some extent	2,000 (20.4%)	661 (19.1%)	638 (29.4%)	377 (31.2%)	800 (26.7%)	
Agree to a large extent	2,437 (24.8%)	633 (18.3%)	654 (30.1%)	293 (24.3%)	908 (30.3%)	
Agree completely	3,070 (31.3%)	1,740 (50.4%)	556 (25.6%)	432 (35.8%)	1,029 (34.3%)	
(2) Alcohol consumption is a personal matter and not something healthcare providers should ask about (“private”)	9,822					
Do not agree	6,548 (66.7%)	2,266 (65.8%)	1,508 (69.5%)	808 (66.9%)	1966 (65.5%)	<0.001
Agree to some extent	2,081 (21.2%)	543 (15.8%)	441 (20.3%)	316 (26.2%)	781 (26.0%)	
Agree to a large extent	665 (6.8%)	274 (8.0%)	166 (7.6%)	43 (3.6%)	182 (6.1%)	
Agree completely	528 (5.4%)	360 (10.5%)	56 (2.6%)	41 (3.4%)	71 (2.4%)	
(3) Healthcare providers should ask about patients’ alcohol consumption, but only if patients seek healthcare to discuss symptoms that could be related to high consumption (“symptoms”)	9,807					
Do not agree	2,300 (23.5)	1,240 (36.2%)	287 (13.2%)	281 (23.3%)	492 (16.4%)	<0.001
Agree to some extent	2,000 (20.4%)	644 (18.8%)	336 (15.5%)	336 (27.8%)	684 (22.8%)	
Agree to a large extent	2,437 (24.8%)	564 (16.5%)	759 (35.0%)	312 (25.8%)	802 (26.7%)	
Agree completely	3,070 (31.3%)	980 (28.6%)	789 (36.3%)	279 (23.1%)	1,022 (34.1%)	
(4) Healthcare providers should ask about patients’ alcohol consumption, but only if the issue is brought up by the patient (“patient”)	9,815					
Do not agree	4,636 (47.2%)	1,595 (46.4%)	993 (45.7%)	577 (47.8%)	1,471 (49.0%)	<0.001
Agree to some extent	2,321 (23.6%)	685 (19.9%)	456 (21.0%)	352 (29.1%)	828 (27.6%)	
Agree to a large extent	1,425 (14.5%)	423 (12.3%)	423 (19.5%)	151 (12.5%)	428 (14.3%)	
Agree completely	1,433 (14.6%)	733 (21.3%)	299 (13.8%)	128 (10.6%)	273 (9.1%)	
(5) I believe people answer honestly when they are asked about their alcohol consumption at healthcare visits (“honesty”)	9,757					
Do not agree	3,609 (37.0%)	1,870 (55.4%)	632 (29.1%)	341 (28.2%)	766 (25.5%)	<0.001
Agree to some extent	3,860 (39.6%)	816 (24.2%)	904 (41.6%)	649 (53.7%)	1,491 (49.7%)	
Agree to a large extent	1,596 (16.4%)	360 (10.7%)	490 (22.6%)	163 (13.5%)	583 (19.4%)	
Agree completely	692 (7.1%)	332 (3.4%)	145 (6.7%)	55 (4.6%)	160 (5.3%)	

### Sociodemographic Variables

Age, gender, and educational level were also collected, with educational level (coded “lower” for high-school graduate or below, and “higher” for college/university education or above).

### Alcohol Consumption

Alcohol consumption was measured using the Alcohol Use Disorders Identification Test (AUDIT) (23) in England and AUDIT-C in Norway, Sweden and the Netherlands. The definition of risky drinking is primarily based on the level of alcohol consumption that is associated with negative consequences, such as health problems, accidents, and social issues. However, the definition of risky drinking varies across countries and might depend on the cultural and social context (24). Risky drinking was analyzed using country-specific definitions of risky drinking (13–17).

(i) In England, the definition of risky drinking was based on the 10 AUDIT questions relating to: alcohol consumption (items 1–3); alcohol dependence (items 4–6) and alcohol-related harm (items 7–10). Based on their responses, two drinking categories were made: lower-risk drinkers (score of <8) or risky drinkers (score of ≥8) (13). One standard drink in England contains 8 g of pure alcohol.
(ii) In Sweden, Norway and the Netherlands, three drinking status categories were constructed based on answers to the first three questions of AUDIT, i.e., the consumption questions (23): abstainers, moderate drinkers, and risky drinkers. These three drinking categories were dichotomised for this study and low risky drinkers (abstainers and moderate drinkers) were compared to risky drinkers. Participants who reported that they had not been drinking any alcohol in the past 12 months were categorised as abstainers. Risky drinking was defined as having a weekly consumption of > nine standard drinks for women in Norway (15) and Sweden (17) and up to 7 drinks in the Netherlands (14); and >14 standard drinks for men and/or engaging in heavy episodic drinking (HED, four standard drinks or more per occasion for women, five for men) monthly in all three countries. These levels used in Norway and Sweden are the recommended levels in Swedish guidelines (25). One standard drink in Sweden or Norway equals 12 g of pure alcohol, and 10 g in the Netherlands.

### Statistical Methods

The distribution of sample characteristics and attitudes towards discussion in healthcare was estimated for each country. Differences in proportions were compared between countries using the chi-squared test, and differences in continuous variables between countries were compared using analysis of variance or Kruskal-Wallis test.

A varimax-rotated principal components factor analysis was performed among the different attitude variables to derive simplified beliefs and attitude components. Two composite dimensions were derived from the factor analysis. The factors identified by the factor analysis were dichotomized at the upper tertile and used as dependent variables in logistic regression. A logistic regression was first performed using the data set with the four countries using country as an independent variable, and odds ratios (OR) were estimated with 95% confidence intervals. The logistic regression analysis was performed using two different models: model I provides crude odds ratios of the following study determinants: age, gender, education, risky drinking, and country; and model II provides odds ratios multivariate adjusted for age, gender, education, risky drinking and country. A test of interaction was performed between study determinants and country using the likelihood test. The interaction was highly statistically significant (*p* < 0.001) both for factors I and II as outcomes, and therefore the logistic regression was performed separately for each country. Results were considered statistically significant at *p* < 0.05 using two-tailed tests. Statistical analyses were performed with SPSS 28 and Stata 17.

## Results

### Participant Characteristics

Table 1 presents sociodemographic and drinking characteristics by country. Alcohol consumption (proportion of adults with episodic drinking and overall alcohol consumption) amongst survey respondents was comparable with official national data sources in each country (1). Patterns of educational level were similar to national data in England and Sweden, but a larger proportion of respondents in Norway (70% vs. 43%) and the Netherlands (63% vs. 37%) were higher educated compared to national data (26). Age and gender of respondents were similar to official data sources in most countries except the Netherlands, where respondents were comparatively older (42% over 60 years vs. 22% aged 65 or more) (27, 28).

Population characteristics from each country regarding age, sex, education, and alcohol consumption were published in previous publications (13–17).

The descriptive statistics for the different attitudes about alcohol prevention in routine healthcare are shown in Table 2. This was presented in previous publications for England, Norway, and Sweden, but not for the Netherlands.

### Composite Dimensions of Attitudes About Alcohol in Routine Healthcare

Factor analysis suggested there were two different factors affecting how alcohol conversations in healthcare are perceived. I. Negative attitude towards discussing alcohol with healthcare providers (including 1. routine, 2. private, 3. symptoms, 4. patient) and II. Perceived honesty and confidence in healthcare (including 1. routine, 5. honesty) (Table 2).

Together, the two derived factors captured 59% of the total variance. The first factor “negative attitudes towards preventive alcohol conversations” captured 38% of the variance. The second factor “Perceived honesty and confidence in healthcare” captured 21% of the variance.

### Negative Attitudes Towards Discussing Alcohol in Routine Healthcare (Factor I)

A high level of education reduced the likelihood of having negative attitudes towards discussing alcohol in healthcare in all countries (OR 0.65, CI 0.59–0.71) (Table 3). Netherlands had the highest OR of negative attitudes (OR 1.50, CI 1.33–1.70) compared to the other countries. Risky drinkers were more likely to report negative attitudes towards discussing alcohol in healthcare in Sweden (OR 1.56, CI 1.32–1.86), Norway (OR 1.61, CI 1.23–2.11), and the Netherlands (OR 1.31, CI 1.08–1.59), but there was no association in England (OR 0.92, CI 0.74–1.15) (Table 5). Women were less likely to report negative attitudes towards discussing alcohol in healthcare, except in the Netherlands where there was no significant association (0.86, CI 0.72-1.03; *p* = 0.10) (Table 5). There was no association between age and negative attitudes towards alcohol discussion in healthcare except in the Netherlands, where those older than 50 were 1.4 more likely to report negative attitudes towards healthcare compared to the younger age group [50–59 years (OR 1.41, CI 1.03–1.95); ≥60 years (OR 1.43, CI 1.08–1.89)] (Table 5).

**TABLE 3:** Logistic regression of having a high level of “Negative attitudes towards discussing alcohol with healthcare providers.” England, Netherlands, Norway, and Sweden, 2017–2018.

Variables	Model I (crude)^a^				Model II (Multivariate)^b^			
	*n* (%)	OR	95% CI	*p*-value	*n* (%)	OR	95% CI	*p*-value
Gender								
Man	4,803	1			4,575	1		
Women	5,073	0.61	0.57–0.64	<0.001	4,866	0.73	0.65–0.71	<0.001
Age								
≤29 years	2,052	1			1,961	1		
30–39 years	1,557	0.98	0.85–1.12	0.738	1,530	0.94	0.72–1.22	0.130
40–49 years	1,672	1.01	0.88–1.16	0.855	1,624	1.04	0.80–1.36	0.112
50–59 years	1,772	1.00	0.87–1.14	0.960	1,723	1.16	0.89–1.52	0.687
≥60 years	2,799	1.16	1.03–1.31	0.017	2,603	1.42	1.04–1.95	0.048
Education								
Basic or secondary school	4,825	1			4,801	1		
University	4,656	0.69	0.64–0.75	<0.001	4,640	0.65	0.59–0.71	<0.001
Drinking categories^c^								
Low risky drinking	7,527	1			7,172	1		
Risky drinking	2,333	1.37	1.25–1.51	<0.001	2,269	1.33	1.21–1.48	<0.001
Country								
Sweden	3,000	1			2,996	1		
Norway	1,208	0.88	0.76–1.01	0.074	1,191	0.94	0.81–1.10	0.455
Netherlands	2,173	1.42	1.26–1.59	<0.001	2,093	1.50	1.33–1.70	<0.001
England	3,499	1.04	0.94–1.15	0.481	3,161	0.99	0.89–1.11	0.889

OR, odds ratios; CI, confidence interval.

^a^ Model I is crude.

^b^ In model II, ORs are multivariate adjusted for gender, age, education, alcohol consumption and country.

^c^ Different definitions used in England, Netherlands, and Norway/Sweden—see methods.

### Perceived Honesty and Confidence Towards Healthcare (Related to Addressing Alcohol) (Factor II)

There was no association between education and the dimension perceived honesty and confidence towards healthcare (OR 0.95, CI 0.87–1.04) (Table 4). Respondents from the Netherlands (OR 0.78, CI 0.69–0.88) and England (OR 0.65, CI 0.59–0.73) displayed lower level of perceived honesty and confidence in healthcare (Table 4). Risky drinkers were more likely to report low perceived honesty and confidence towards healthcare in all four countries compared to lower risk drinkers (OR 0.70, CI 0.63–0.78) (Table 4). Women reported lower levels of the dimension perceived honesty and confidence towards healthcare compared with men in England (0.68, CI 0.59–0.80; *p* < 0.001) and in Norway (0.69, CI 0.54–0.88; *p* = 0.003). In Sweden (0.87, CI 0.75–1.01; *p* = 0.069) and the Netherlands (0.91, CI 0.71–1.05; *p* = 0.137) there was no statistically significant association between gender and perceived honesty and confidence towards healthcare (Table 6). Being older than 60 years increased the OR for the dimension perceived honesty and confidence towards healthcare to 1.62 times (CI 1.21–2.18) in the Netherlands, while it decreased it to 0.79 times (CI 1.08–1.89) in England (Table 6).

**TABLE 4:** Logistic regression of having a high level of “Perceived honesty and confidence in healthcare.” England, Netherlands, Norway, and Sweden, 2017–2018.

Variables	Model I (crude)^a^				Model II (Multivariate)^b^			
	*n* (%)	OR	95% CI	*p*-value	*n* (%)	OR	95% CI	*p*-value
Gender								
Man	4,803	1			4,575	1		
Women	5,073	0.80	0.74–0.87	<0.001	4,866	0.79	0.73–0.87	<0.001
Age								
≤29 years	2,052	1			1,961	1		
30–39 years	1,557	0.87	0.75–0.99	0.043	1,530	0.85	0.74–0.98	0.029
40–49 years	1,672	0.83	0.73–0.96	0.010	1,624	0.82	0.71–0.95	0.007
50–59 years	1,772	0.89	0.78–1.02	0.082	1,723	0.88	0.77–1.01	0.070
≥60 years	2,799	0.97	0.86–1.10	0.664	2,603	1.03	0.91–1.17	0.625
Education								
Basic or secondary school	4,825	1			4,801	1		
University	4,656	0.97	0.89–1.05	0.467	4,640	0.95	0.87–1.04	0.293
Drinking categories^c^								
Low risky drinking	7,527	1			7,172	1		
Risky drinking	2,333	0.78	0.70–0.86	<0.001	2,269	0.70	0.63–0.78	<0.001
Country								
Sweden	3,000	1			2,996	1		
Norway	1,208	0.90	0.78–1.03	0.124	1,191	0.91	0.79–1.05	0.211
Netherlands	2,173	0.80	0.71–0.90	<0.001	2,093	0.78	0.69–0.88	<0.001
England	3,499	0.73	0.66–0.81	<0.001	3,161	0.65	0.59–0.73	<0.001

OR, odds ratios; CI, confidence interval.

^a^ Model I is crude.

^b^ In model II, ORs are multivariate adjusted for gender, age, education, alcohol consumption and country.

^c^ Different definitions used in England, Netherlands and Norway/Sweden—see methods.

## Discussion

This study aimed to explore how educational levels, as a proxy measure of socio-economic status, are associated with attitudes towards alcohol conversations in healthcare using population-based surveys in England, the Netherlands, Norway, and Sweden, and to compare patterns between countries. We found similarities and differences between countries in the extent to which respondents were more or less supportive of alcohol conversations in routine healthcare according to individual characteristics such as education, gender, age, and alcohol consumption.

In all countries, men, and respondents with a low level of education, were significantly more negative towards discussing alcohol in routine healthcare consultations compared to other groups. Perceived honesty and confidence in healthcare was lower among women in Norway and England, among older respondents in England, and among risky drinkers in all countries. However, there were no clear differences by level of education. Respondents from the Netherlands reported higher levels of negative attitudes towards discussing alcohol in healthcare compared to other countries and along with those from England, displayed lower level of perceived honesty and confidence in healthcare overall.

One important finding concerned the association between lower levels of educational attainment and negative attitudes towards alcohol conversations in healthcare, which is consistent with previous research (11–13). At the same time, there is also evidence that primary healthcare providers are more likely to ask patients in a lower SES about their drinking compared to those with a higher SES (7, 29), and may more easily identify signs of high alcohol consumption in patients with a lower SES than those of similar SES to themselves (30, 31). Our findings therefore highlight a dilemma: even though evidence suggests that persons with a lower SES are more likely to be asked about alcohol, which could help address health inequalities, this group are more likely to hold negative attitudes towards discussing alcohol with their healthcare provider. One key question is whether these negative attitudes affect the impact of the conversations and/or patients’ engagement with their healthcare professional. In that respect, the manner in which the conversation about alcohol is conducted is important, with previous research suggesting a need for healthcare professionals to demonstrate empathy and promote a non-judgemental atmosphere (15). Furthermore, patients with a low SES, might have lower health literacy skills (21), meaning that health professionals should ensure they discuss alcohol in an accessible and understandable way.

There was no association between educational level and the dimension “Perceived honesty and confidence in healthcare” in any of the countries at the individual level (Factor II). Individuals with more harmful drinking habits are less likely to report their actual alcohol consumption both among low and high educated.

Women were more likely to be supportive towards alcohol discussion in healthcare (Factor I), and this was significant in all countries except in the Netherlands. This is consistent with results of a previous study that showed that women had more positive attitudes towards health promotion (32, 33). Possible explanations for the fact that women had more positive attitude towards alcohol prevention than men might be better experience of patient-physician relation among women (women have a higher healthcare consumption compared to men) and the fact that there is a lower prevalence of risky drinkers among women (34). At the same time, we found that women had lower “perceived honesty and confidence in healthcare” (Factor II) in Norway and England; it was not significant in Sweden and in the Netherlands. An explanation for this association could be a possible gender bias in healthcare (35). Another explanation may be that even though women visit healthcare twice as often as men, men have twice as many conversations about alcohol as women (36). Harmful alcohol consumption may be more difficult to identify in women than men, due to the fact that alcohol abuse is more stigmatized among women than men (37).

In the Netherlands, those older than 50 years were more likely to report negative attitudes towards alcohol conversations in healthcare compared to the younger age groups [50–59 years (OR 1.41 CI 1.03–1.95); ≥60 years (OR 1.43, CI 1.08–1.89)] (Table 5). Being older than 60 years was also associated with a lower perceived honesty and confidence towards healthcare in England (Table 6). This is consistent with previous results from England (13), where older participants were more reserved towards alcohol prevention compared to younger participants, and data suggested that healthcare providers asked older adults about their drinking less frequently. At the same time, in the Netherlands, being older than 60 years increased the odds ratio for perceived honesty and confidence towards healthcare to 1.62 (CI 1.08–1.89) compared to the young (Table 6), which is consistent with previous findings from Sweden and Norway where older participants were more positive towards alcohol prevention in healthcare (12, 16). This complex association between age and attitudes towards alcohol prevention in healthcare is evident in previous research which found that older adults favoured policies that restrict alcohol use in public places, whereas younger adults favoured an increase in alcohol taxes to address underage alcohol use (32). Additionally, the differences in the association between age and attitudes towards alcohol prevention in healthcare might be due to cultural differences between countries, as well as variations in how healthcare is organised and delivered (38).

**TABLE 5:** Logistic regression of having a high level of “Negative attitudes towards discussing alcohol in healthcare” stratified by country. England, Netherlands, Norway, and Sweden, 2017–2018.

Variables	Country (*n* = 9,441)											
	Sweden (*n* = 2,996)			Norway (*n* = 1,191)			Netherlands (*n* = 2093)			England (*n* = 3,161)		
	OR^a^	95% CI	*p*-value	OR^a^	95% CI	*p*-value	OR^a^	95% CI	*p*-value	OR^a^	95% CI	*p*-value
Gender												
Man	1			1			1			1		
Women	0.56	0.48–0.66	<0.001	0.75	0.58–0.97	0.027	0.86	0.72–1.03	0.102	0.84	0.72–0.97	0.020
Age												
≤29 years	1			1			1			1		
30–39 years	1.08	0.85–1.35	0.533	1.00	0.63–1.58	0.996	1.37	0.96–1.95	0.085	1.07	0.83–1.39	0.592
40–49 years	1.15	0.92–1.44	0.223	1.04	0.67–1.61	0.866	1.12	0.78–1.58	0.544	1.11	0.86–1.43	0.418
50–59 years	1.06	0.85–1.34	0.599	0.83	0.53–1.29	0.407	1.41	1.03–1.95	0.035	0.86	0.66–1.11	0.229
≥60 years	1.12	0.85–1.48	0.426	1.00	0.66–1.52	0.982	1.43	1.08–1.89	0.012	1.01	0.81–1.24	0.936
Education												
Basic or secondary school	1			1			1			1		
University	0.73	0.62–0.85	<0.001	0.55	0.42–0.72	<0.001	0.66	0.56–0.80	<0.001	0.63	0.53–0.74	<0.001
Drinking categories^b^												
Low risky drinking	1			1			1			1		
Risky drinking	1.56	1.32–1.86	<0.001	1.61	1.23–2.11	<0.001	1.31	1.08–1.59	0.007	0.92	0.74–1.15	0.479

OR, odds ratios; CI, confidence interval.

^a^ ORs are multivariate adjusted for gender, age, education, and alcohol consumption.

^b^ Different definitions used in England, Netherlands, and Norway/Sweden—see methods.

**TABLE 6:** Logistic regression of having a high level of “Perceived honesty and confidence in healthcare” stratified by country. England, Netherlands, Norway, and Sweden, 2017–2018.

Variables	Country											
	Sweden (*n* = 2,996)			Norway (*n* = 1,191)			Netherlands (2093)			England (*n* = 3,161)		
	OR^a^	95% CI	*p*-value	OR^a^	95% CI	*p*-value	OR^a^	95% CI	*p*-value	OR^a^	95% CI	*p*-value
Gender												
Man	1			1			1			1		
Women	0.87	0.75–1.01	0.069	0.69	0.54–0.88	0.003	0.91	0.71–1.05	0.137	0.68	0.59–0.80	<0.001
Age												
≤29 years	1			1			1			1		
30–39 years	0.79	0.64–0.99	0.037	0.66	0.43–1.02	0.060	0.87	0.59–1.29	0.494	1.05	0.81–1.36	0.729
40–49 years	0.70	0.56–0.87	0.001	0.59	0.39–0.89	0.013	1.14	0.79–1.65	0.486	1.03	0.79–1.33	0.848
50–59 years	0.92	0.75–1.15	0.478	0.57	0.38–0.88	0.010	1.23	0.88–1.74	0.230	0.84	0.65–1.08	0.169
≥60 years	1.19	0.92–1.55	0.184	0.88	0.60–1.29	0.507	1.62	1.21–2.18	0.001	0.79	0.64–0.98	0.035
Education												
Basic or secondary school	1			1			1			1		
University	1.00	0.86–1.16	0.949	0.99	0.76–1.29	0.933	0.86	0.71–1.05	0.137	0.96	0.81–1.13	0.627
Drinking categories^b^												
Low risky drinking	1			1			1			1		
Risky drinking	0.70	0.59–0.83	<0.001	0.71	0.54–0.93	0.012	0.74	0.60–0.91	0.005	0.62	0.49–0.79	<0.001

OR, odds ratios; CI, confidence interval.

^a^ ORs are multivariate adjusted for gender, age, education, and alcohol consumption.

^b^ Different definitions used in England, Netherlands, and Norway/Sweden—see methods.

Risky drinking was associated with negative attitudes towards alcohol conversations in healthcare in all countries, except England. This is consistent with a previously published Swedish study, which found lower levels of support for routinely asking patients about their alcohol consumption amongst excessive and hazardous drinkers (12). Individuals engaged in detrimental alcohol habits display a preference towards limited public commitment to improving their health (33). Other research on alcohol consumption also points to a negative association between risky drinking and support for alcohol prevention, with heavy drinkers being less supportive of interventions in general (12, 32). The difference observed in England is consistent with a previously published study (13) and might be explained by cultural differences between England and the three other countries. Risky drinking was also associated with significant low level of “perceived honesty and confidence in healthcare” (Factor II) in all four countries. The fact that risky drinkers have lower “perceived honesty and confidence in healthcare” (Factor II) shows that this dimension includes not only individual attitudes but also contextual structural factors (39).

### Comparison Between Countries

We found similarities in attitudes towards alcohol conversation in healthcare across England, Norway, Sweden and the Netherlands. People with a lower educational attainment had a higher level of negative attitudes, and risky drinkers had less perceived honesty and confidence in healthcare in all countries. However, Sweden and Norway had the highest level of positive attitudes towards alcohol conversations as routine, and a higher perceived honesty and confidence in healthcare.

There were also differences between countries in attitudes towards alcohol conversation in healthcare. Respondents in the Netherlands were significantly more likely to have negative attitudes towards alcohol prevention compared to those from other countries (OR = 1.5 compared to Sweden), and Norway and England were like Sweden (Table 3). The level of perceived honesty in healthcare was also significantly lower in the Netherlands (OR = 0.78) and in England (OR = 0.65) compared to Sweden and Norway (Table 4). Based on findings from a previous study comparing differences in public trust in healthcare between Germany, the Netherlands, England and Wales, the varied attitudes towards alcohol conversations we observed between countries might result from a combination of cultural differences and differences in how healthcare is organized (38).

### Strengths and Limitations

The study has several strengths. The data sets are large and similar population-based surveys were performed in four different countries. This is the first study to include four countries in a study of attitudes in addressing alcohol conversations in healthcare. This research is valuable to inform preventative alcohol policies in the future and to improve strategies to tackle the higher alcohol-related morbidity and mortality in lower socio-economic groups in future.

The study has several limitations that are important to consider. In the Netherlands and Norway, the study samples had more educated respondents compared to national population estimates, which might have reduced the association between attitudes alcohol prevention and education. Furthermore, the original studies used different definitions of risky drinking between countries. The study used different educational coding systems in each country, necessitating that the 7-level variable on education had to be reduced to two-levels when comparing the countries. In England, the higher level includes only university education. Despite this loss of information, the study was able to detect large difference between low and high educated respondents in attitudes towards alcohol prevention in healthcare.

### Conclusion

We identified socio-demographic and behavioural determinants of attitudes towards discussing alcohol in healthcare in four European countries, with some key similarities and differences between countries. The results suggest that trust in healthcare is an important underlying mechanism of attitudes towards alcohol conversations in healthcare. We also found a higher likelihood of having negative attitudes towards discussing alcohol in healthcare among respondents with low education in all four countries, as well as lower perceived honesty and confidence in healthcare among risky drinkers.

### Implications and Future Research

Our findings suggest that the implementation of alcohol prevention in healthcare targeting all patient groups has resulted in inequalities in attitudes towards discussion about alcohol in healthcare. Respondents with low education and risky drinkers are more negative towards routine delivery of alcohol prevention activities in healthcare settings. This highlights the need for more public information on alcohol prevention together with better training on alcohol issues for healthcare professionals to ensure that discussions about alcohol preventative measures are adapted to all patient groups. Given the evidence of increasing social inequalities in health (20), a stronger commitment is needed from the authorities in reducing alcohol consumption in the population. The results of this study provide insights into the complex relationship between alcohol and culture, which means that policy interventions to address alcohol-related harms should be adapted to each country.

Future research to further explore patients’ experiences and views towards alcohol conversations in healthcare is warranted, as well as the development of more diverse methods to implement preventive measures in healthcare. Less educated and risky drinkers have more negative attitudes: this implies that this should be studied further. Sociodemographic factors should be considered when implementing alcohol prevention in healthcare.
